# Embolic Stroke due to a Common Carotid Artery Thrombus in a Young Patient with Severe Iron-Deficiency Anemia without Thrombocytosis

**DOI:** 10.1155/2016/6920303

**Published:** 2016-09-26

**Authors:** David Roshal

**Affiliations:** Department of Neurology, Kennedy University Hospital, Stratford, USA

## Abstract

This case report describes a 41-year-old previously healthy male who presented with stuttering transient ischemic symptoms and radiographic evidence of a left common carotid artery thrombus as well as acute and subacute ischemic infarcts in the left middle cerebral artery territory. An exhaustive stroke work-up did not provide a plausible etiology for his symptoms. His complete blood count and iron studies, however, revealed evidence of severe iron-deficiency anemia without reactive thrombocytosis. His stool guaiac test was positive. He was discharged home on oral antithrombotic agents and aggressive iron replacement therapy with a plan for repeat vascular imaging in 3 months and a colonoscopy. This case report suggests that severe iron-deficiency anemia with or without reactive thrombocytosis should be viewed as a possible hematologic condition associated with thrombotic tendencies and a risk factor for ischemic stroke, especially in young adults. Aggressive iron supplementation and short-term antithrombotic therapy with follow-up vascular imaging are a reasonable treatment for these patients.

## 1. Introduction

Few cases of carotid artery thrombus associated with severe iron-deficiency anemia and reactive thrombocytosis have been reported in young adults [1–4]; however, normal platelet counts in these patients have rarely been described. The pathogenic mechanisms leading to thrombus formation and ischemic stroke due to severe iron-deficiency anemia are not well understood, especially in patients without thrombocytosis. A case report is presented here that reviews the potential causes for the suspected hypercoagulable state produced in young patients with ischemic stroke related to severe iron-deficiency anemia.

## 2. Case Presentation

The patient is a 41-year-old man without vascular risk factors who developed acute stuttering right upper extremity weakness and paresthesias associated with mild slurred speech and right facial droop. He also described having several episodes of right upper extremity numbness and tingling intermittently over that past month. He denied any recent head or neck trauma. His past medical history was remarkable for rectal bleeding. He was taking no medications. Family history was negative for neurological or hematologic diseases.

**Figure 1:**
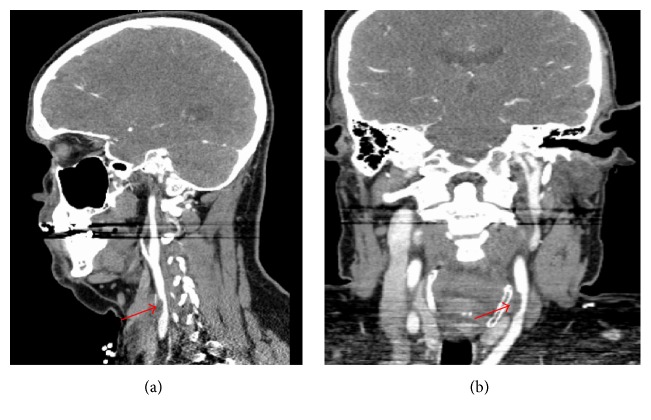
Sagittal (a) and coronal (b) CT angiogram of the head and neck demonstrating a 1.5 × 0.8 cm focal adherent mural thrombus (red arrows) in the left common carotid artery 4 cm proximal to the bifurcation resulting in less than 50% stenosis of the luminal diameter.

He was afebrile and blood pressure was 139/82 mm Hg with a heart rate of 54 beats per minute that was regular. The general physical examination revealed conjunctival pallor. The patient's stool was hemoccult-positive. His initial neurological examination revealed a National Institutes of Health Stroke Scale (NIHSS) of 2, which included a mild right lower facial droop and upper extremity pronator drift. Given his mild deficits he did not receive intravenous tissue plasminogen activator. The patient was admitted to the intensive care unit for close neurological observation. His noncontrast head CT (not shown) revealed an old right frontal wedge-shaped embolic ischemic infarct. Laboratory analysis was remarkable for a microcytic hypochromic anemia with normal platelet count (see below). A CT angiogram of the head and neck showed a 1.5 × 0.8 cm thrombus adhering to the wall of the left common carotid artery 4 cm proximal to the bifurcation without evidence of atherosclerosis or dissection (Figure 1). MRI of the brain (not shown) revealed acute and subacute embolic small volume cortical infarcts in the left middle cerebral artery territory as well as an old right frontal wedge-shaped moderate volume cortical embolic infarct.

The white blood cell count was 6,200/*μ*L with normal differential, hemoglobin 8.9 g/dL, hematocrit 30.8%, mean corpuscular volume 59.6 (80–96.5 fL), and platelets 355,000/*μ*L (150,000–400,000). The peripheral smear showed presence of red blood cell hypochromia and microcytosis. Serum iron was 14 *μ*g/dL (45–160), ferritin 2.1 ng/mL (23.9–336.2), total iron binding capacity 480 *μ*g/dL (228–428), and transferrin saturation 3% (20–50). He had mild hyperlipidemia. Results of additional tests were all normal, including comprehensive hypercoagulable (prothrombin G20210A, Factor V Leiden, antithrombin III, anti-cardiolipin antibody IgG, IgM, and IgA, lupus anticoagulant, homocysteine, Protein C/S and Factor VIII activity, MTHFR mutation, anti-beta 2 glycoprotein antibody IgG, IgM, and IgA), rheumatological, and chemistry panels, hemoglobin A1C, urine drug screen, serum protein electrophoresis, prothrombin time, and partial thromboplastin time. Electrocardiogram and chest X-ray were normal. Transesophageal echocardiogram (TEE) was normal except for an incidental small patent foramen ovale in the resting state. Lower-extremity venous Doppler and pelvic magnetic resonance venography failed to detect any source of thrombus. CT of the abdomen and pelvis with oral and intravenous contrast did not detect any malignant disease.

**Figure 2:**
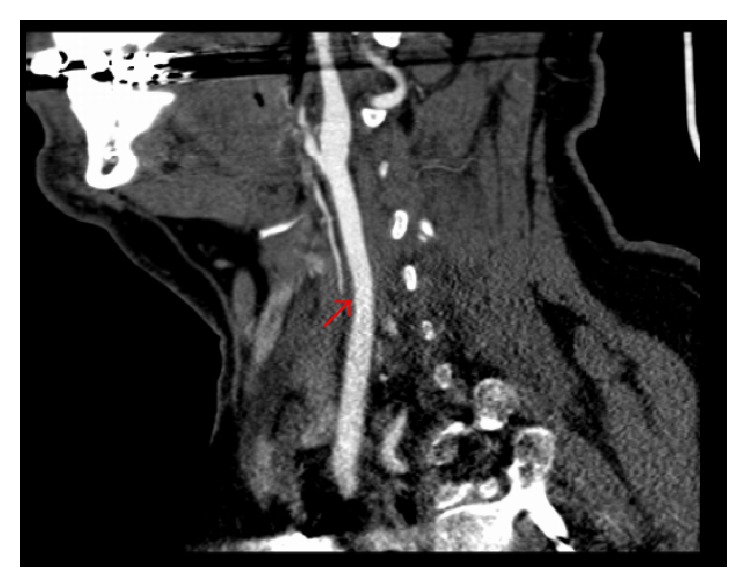
Sagittal CT angiogram of the neck (3 months later) showing very minimal mural thrombus remaining.

The severe iron-deficiency in this case was attributed to a gastrointestinal source. The patient received intravenous iron replacement and was bridged from heparin to warfarin with the aim of achieving an International Normalization Ratio (INR) of 2 to 3. He was discharged home without any deficits on low dose aspirin and warfarin as well as oral iron replacement therapy with a plan for repeat vascular imaging in 3 months, hematology follow-up, a colonoscopy, and a 30-day cardiac event monitor. His repeat CT angiogram 3 months later (Figure 2) showed very minimal mural thrombus remaining and therefore his anticoagulation was discontinued; however, he remained on a low dose aspirin. His 30-day cardiac event monitor had not revealed any paroxysmal atrial fibrillation or flutter. He has continued to do well without any recurrent ischemic embolic events.

## 3. Discussion

The finding of a mural thrombus involving the common carotid artery is extremely uncommon because of the rarity of significant atherosclerotic disease in this large diameter and nonturbulent vessel [1–4]. It is not clear why severe iron-deficiency anemia is associated with thrombus formation in the carotid arteries in the absence of vascular disease [1–4]. Various pathogenic mechanisms have been proposed including altered hemodynamics resulting in increased turbulent flow and subsequent endothelial injury, reactive thrombocytosis due to elevated erythropoietin levels, and enhanced platelet aggregation and function caused by elevated erythropoietin levels, increased oxidant stress, and reduced activity of iron-containing platelet monoamine oxidase that catabolizes the serotonin necessary for platelet function [5–8].

In 1983, Alexander first reported that a patient developing a right hemiparesis and aphasia was found to have severe iron-deficiency anemia and marked thrombocytosis [9]. Akins et al. reported 3 case reports in which young women with severe iron-deficiency anemia and thrombocytosis secondary to menorrhagia developed carotid artery thrombosis [1]. Batur Caglayan et al. reported a young woman with severe iron-deficiency anemia due to menorrhagia with transient ischemic attacks and an intraluminal carotid artery thrombus [2]. Idbaih et al. have also reported on 8 patients with spontaneous thrombosis of lesion-free carotid arteries and half of the patients with spontaneous thrombus had severe iron-deficiency anemia, mostly due to menorrhagia [10]. Nakamizo et al. reported a young woman with severe iron-deficiency anemia and thrombocytosis secondary to menorrhagia, who presented with left hemiparesis and was found to have acute right hemispheric infarcts with mural thrombus in a normal left ventricle and carotid artery [4]. Bukharovich et al. reported two young females with history of severe iron-deficiency anemia with reactive thrombocytosis due to menorrhagia presenting with acute arterial embolism and large mobile aortic thrombus in the absence of atherosclerosis [11]. Interestingly, there have also been several case reports of cerebral venous sinus thrombosis and ischemic stroke in the pediatric population being attributed to severe iron-deficiency anemia [6]. The above case reports do not clearly demonstrate causality between severe iron-deficiency anemia and thrombosis; however, they highlight the growing evidence for a potential link between severe iron-deficiency anemia and ischemic stroke.

The pieces of information from previous case reports taken together with the clinical characteristics of this particular patient suggest that severe iron-deficiency anemia likely played a causative role for his ischemic strokes (old and new) by enhancing platelet aggregation with subsequent in situ arterial thrombosis and distal embolism. Although this particular patient had a small patent foramen ovale detected on TEE, emboli from the heart, regardless of etiology, would be expected to travel distally and not lodge in the common carotid artery.

## 4. Conclusion

Severe iron-deficiency anemia with or without reactive thrombocytosis should be viewed as a potential hematologic condition associated with thrombotic tendencies and a risk factor for ischemic stroke, especially in young adults. Aggressive iron supplementation and short-term antithrombotic therapy with follow-up vascular imaging are a reasonable treatment for these patients [2, 12]. Complete dissolution of the mural thrombus without further neurologic progression occurs in the majority of patients treated with heparin to warfarin for several weeks to months. Some experts have added aspirin or other antiplatelet agents to anticoagulation therapy, whereas others used antiplatelet agents alone; however, there are no randomized trials to support this [13]. Although the relationship between severe iron-deficiency anemia and ischemic stroke remains to be further elucidated, patients found to have severe iron-deficiency anemia should be aggressively surveyed and managed for the possible bleeding source in order to reduce the risk of subsequent ischemic stroke.
